# Tumour necrosis factor α regulates the miR‐27a‐3p–Sfrp1 axis in a mouse model of osteoporosis

**DOI:** 10.1113/EP090311

**Published:** 2024-05-15

**Authors:** Dang‐Feng Zhang, Xiao‐Na Jin, Xing Ma, Yu‐Sheng Qiu, Wei Ma, Xing Dai, Zhi Zhang

**Affiliations:** ^1^ Department of Orthopedics The First Affiliated Hospital of Xi'an Jiaotong University Xi'an Shaanxi China; ^2^ Department of Nursing Xi'an International University Xi'an Shaanxi China

**Keywords:** BMSCs, miR‐27a‐3p, osteoporosis, Sfrp1, TNF‐α

## Abstract

Osteoporosis is a metabolic bone disease that involves gradual loss of bone density and mass, thus resulting in increased fragility and risk of fracture. Inflammatory cytokines, such as tumour necrosis factor α (TNF‐α), inhibit osteogenic differentiation of bone marrow mesenchymal stem cells (BMSCs), and several microRNAs are implicated in osteoporosis development. This study aimed to explore the correlation between TNF‐α treatment and miR‐27a‐3p expression in BMSC osteogenesis and further understand their roles in osteoporosis. An osteoporosis animal model was established using ovariectomized (OVX) mice. Compared with Sham mice, the OVX mice had a significantly elevated level of serum TNF‐α and decreased level of bone miR‐27a‐3p, and in vitro TNF‐α treatment inhibited miR‐27a‐3p expression in BMSCs. In addition, miR‐27a‐3p promoted osteogenic differentiation of mouse BMSCs in vitro, as evidenced by alkaline phosphatase staining and Alizarin Red‐S staining, as well as enhanced expression of the osteogenic markers Runx2 and Osterix. Subsequent bioinformatics analysis combined with experimental validation identified secreted frizzled‐related protein 1 (Sfrp1) as a downstream target of miR‐27a‐3p. Sfrp1 overexpression significantly inhibited the osteogenic differentiation of BMSCs in vitro and additional TNF‐α treatment augmented this inhibition. Moreover, Sfrp1 overexpression abrogated the promotive effect of miR‐27a‐3p on the osteogenic differentiation of BMSCs. Furthermore, the miR‐27a‐3p–Sfrp1 axis was found to exert its regulatory function in BMSC osteogenic differentiation via regulating Wnt3a–β‐catenin signalling. In summary, this study revealed that TNF‐α regulated a novel miR‐27a‐3p–Sfrp1 axis in osteogenic differentiation of BMSCs. The data provide new insights into the development of novel therapeutic strategies for osteoporosis.

## Abstract (highlights)

- **What is the central question of this study?**
  What is the mechanism by which TNF‐α plays its critical role in osteogenesis via the modulation of bone loss and formation?
- **What is the main finding and its importance?**
  TNF‐α regulates a novel miR‐27a‐3p–Sfrp1 axis in osteogenic differentiation of bone marrow mesenchymal stem cells.

## 1 INTRODUCTION

Osteoporosis is a progressive metabolic bone disease involving the gradual loss of bone density and mass, resulting in increased fragility and risk of fracture (Akkawi & Zmerly, 2018; Lorentzon, 2019). Even though current therapeutic strategies can reduce the risk of developing osteoporotic fractures, osteoporosis often develops without diagnosis until a fracture occurs (Mauck & Clarke, 2006). Accumulating studies have shown that high levels of inflammatory cytokines could increase the activity of osteoclasts and impair bone formation in osteoporosis (Khosla et al., 2008; Lacativa & Farias, 2010). Specifically, the inflammatory cytokine tumour necrosis factor α (TNF‐α) has been demonstrated to play a critical role in osteogenesis via the modulation of bone loss and bone formation (Yang et al., 2013; Zhao et al., 2011). In accord with these findings, several studies recently suggested that inhibition of TNF‐α‐mediated impairments in osteogenesis, such as using resveratrol, quercetin and artemisinin, can help to promote the osteogenesis of mesenchymal stem cells thereby potentially improved osteoporosis (Hu et al., 2021; Yuan et al., 2020, 2018). However, the underlying mechanism of impaired osteogenic differentiation by inflammatory cytokines remains unclear.

MicroRNAs (miRNAs) are a group of short non‐coding RNAs which participate in various biological processes via post‐transcriptional regulation of target genes (O'Brien et al., 2018). An increasing number of studies have suggested that miRNAs play important regulatory roles in bone marrow mesenchymal stem cell (BMSC) proliferation, differentiation and function (Lei et al., 2011; Shao, 2017; Teng et al., 2016; Zhang et al., 2020). MicroRNA profiling of the entire trabecular bone has identified multiple dysregulated miRNAs associated with osteoporosis (De‐Ugarte et al., 2015). For example, miR‐133a was reported to be upregulated during osteoclastogenesis and promoted osteoclast differentiation of THP‐1 and RAW264.7 cells (Li et al., 2018). In another study, miR‐7b‐5p was found to inhibit the adipogenic differentiation of human BMSCs via insulin receptor substrate 2 (IRS2) targeting, thus alleviating the development of osteoporosis (Li et al., 2019b). In contrast, Cheng et al. reported that miR‐365‐5p could promote the development and progression of osteoporosis via the negative regulation of Runx2 and inhibition of osteogenic differentiation (Cheng et al., 2019). Thus, further dissection of the functional roles of miRNAs in osteoporosis is an important endeavour.

Among the miRNAs involved in osteogenesis, miR‐27a‐3p was found to be associated negatively with the osteogenic differentiation of MC3T3‐E1 pre‐osteoblasts by targeting Osterix (Xu et al., 2020). In addition, exosome‐derived miR‐27a‐3p from C2C12 myoblasts could enhance the osteogenic differentiation of MC3T3‐E1 pre‐osteoblasts (Xu et al., 2018). Another study demonstrated that long non‐coding RNA MEG3 promoted osteogenic differentiation of periodontal ligament stem cells through the miR‐27a‐3p–insulin‐like growth factor 1 (IGF1) axis in periodontitis (Liu et al., 2019b). However, it is unclear whether miR‐27a‐3p functions as a downstream regulator of inflammatory signals and participates in the TNF‐α‐mediated osteoporosis. Therefore, we focused on the correlation between TNF‐α stimulation and miR‐27a‐3p expression to clarify the role of miR‐27a‐3p in the development of osteoporosis.

Secreted frizzled‐related protein 1 (Sfrp1) is a member of the Sfrp family regulating the Wnt signalling pathway (Bovolenta et al., 2008). Downregulation of Sfrp1 has been reported in colorectal cancer, and low expression levels of Sfrp1 have been associated with poor prognosis in colorectal cancer patients (Huang et al., 2014). In addition, Sfrp1 has been reported to negatively regulate BMSC proliferation and differentiation (Hu et al., 2019; Tang et al., 2019). However, the exact mechanisms underlying the regulation of Sfrp1 expression and the role that Sfrp1 plays in regulating osteogenic differentiation of BMSCs remain unknown.

In this study, we used the ovariectomized (OVX) mouse model and demonstrated that elevated levels of TNF‐α suppressed the expression of miR‐27a‐3p during osteogenic differentiation of BMSCs. MiR‐27a‐3p promoted osteogenic differentiation and exerted its function through the negative regulation of Sfrp1 and enhancement of β‐catenin–C‐Myc–Cyclin D1 signalling. These results suggest a novel regulatory axis of miR‐27a‐3p and Sfrp1 in modulating the osteogenic differentiation of BMSCs, which provides critical insights into the clinical diagnosis and treatment of osteoporosis.

## 2 METHODS

### 2.1 Ethical approval

All animal experiments were approved by the Experimental Animal Ethics Committee of Xi'an Jiaotong University (Shaanxi, China; approval No. XJTULAC2019559‐17). All animal experiments followed the *Guide for the Care and Use of Laboratory Animals* (2011 Eighth Edition, National Research Council) for the welfare of the laboratory animals. All necessary procedures were implemented to minimize the pain and suffering of animals.

### 2.2 Cell culture and transfection

HEK293T cells were obtained from the Cell Bank of Type Culture Collection of Chinese Academy of Sciences (Shanghai, China) and cultured in Dulbecco's modified Eagle's medium (Thermo Fisher Scientific, Waltham, MA, USA) which was supplemented with 10% fetal bovine serum (FBS; Thermo Fisher Scientific), 1% penicillin, and streptomycin (Thermo Fisher Scientific) in a 37°C incubator with 5% CO_2_. Transfection of miRNA mimics, inhibitors and plasmids was performed using Lipofectamine 3000 (Thermo Fisher Scientific) according to the manufacturer's protocol. Specifically, miR‐27a‐3p mimics (5ʹ‐TTCACAGTGGCTAAGTTCCGC‐3ʹ), miR‐27a‐3p inhibitor (5ʹ‐GCGGAACTTAGCCACTGTGAA‐3ʹ) and the negative control (5ʹ‐TTGTACTACACAAAAGTACTG‐3ʹ) were purchased from Thermo Fisher Scientific. Small hairpin RNAs (shRNA)‐targeting Sfrp1 and scramble control were from GenePharma (Shanghai, China). The full‐length sequence of Sfrp1 open reading frame was amplified and cloned into a pcDNA3.1 vector (Thermo Fisher Scientific) to generate the overexpression vector termed pcDNA3.1‐Sfrp1. Plasmid construction, preparation and verification by DNA sequencing were performed by GenePharma.

### 2.3 OVX model procedures

Wild‐type C57BL/6 female mice were purchased from Vital River Laboratory (Beijing, China) and were randomly divided into two groups: the Sham group (*n* = 5) and OVX group (*n* = 5). The OVX group underwent bilateral ovariectomy by the dorsal approach. After adaptive housing for 1 week, the OVX modelling was performed as previously reported (Li et al., 2019a). Briefly, anaesthesia was performed using 1.5% isoflurane at a flow rate in the range of 0.5–1.0 L/min. Then, hair in the dorsal mid‐lumbar area was shaved and the skin was cleaned to make a 20‐mm midline dorsal skin incision. Following this, the paired ovaries were excised and the wound was closed using suturing. Mice in the Sham group received the same procedure except ovary excision. The post‐operative analgesia was performed by adding 1.7 μg/mL meloxicam into the drinking water. Since the entire operation and animal maintenance was performed in a pathogen‐free animal facility, no antibiotic was used in this study. Eight weeks later, mice were killed with an overdose of isoflurane (5% isoflurane at a rate of 1.0 L/min for 20 min). The killing was confirmed with cardiac and respiratory arrest. Subsequently, the serum and leg samples were harvested and stored at –80°C until utilized.

### 2.4 BMSC isolation, characterization and treatment

Mouse BMSCs were obtained from long bones of the indicated C57BL/6J mice. Mice were killed, and bone marrow cells were collected by flushing nucleate cells out of the femurs and tibiae with cold phosphate buffered saline (PBS). Cells were cultured (2 × 10^6^ nucleated cells/10 cm dish) in α‐Minimum Essential Medium (Thermo Fisher Scientific) supplemented with 10% FBS (Thermo Fisher Scientific), 1% penicillin–streptomycin in an incubator at 37°C with 5% CO_2_. Three days later, non‐adherent cells were removed. The adhering cells were cultured in intact medium and changed every 2 days. After one passage, the primary BMSCs were cryopreserved in liquid nitrogen using a cryopreservation/recovery kit (AccuRef Scientific, Xi'an, China). When recovered, the BMSCs were passaged at least once and phenotypically confirmed by flow cytometry before an experiment.

The purity of BMSCs was analysed by flow cytometry. Briefly, the cultured mouse BMSCs were digested and resuspended in PBS which contained 2% FBS. The cells (2 × 10^5^ cells/test) were incubated with phycoerythrin (PE)‐conjugated antibodies for 30 min in 100 μL PBS containing 2% FBS. After rinsing with PBS three time, the cells were resuspended with 200 μL PBS + 2% FBS and analysed by a flow cytometer (BD Biosciences, San Jose, CA, USA). The antibodies were the following: PE‐conjugated rat anti‐mouse CD73 (cat. no. 550741; BD Biosciences); PE‐conjugated rat anti‐mouse CD105 (cat. no. 562762; BD Biosciences), PE‐conjugated rat anti‐mouse CD90.1 (cat. no. 561404; BD Biosciences), PE‐conjugated rat anti‐mouse CD34 (cat. no. 551387; BD Biosciences), and CD45 (cat. no. 562420; BD Biosciences). Mouse IgG isotype was used as the control (cat. no. 565832; BD Biosciences). A BMSC purity >97% was considered appropriate, and the purity‐confirmed BMSCs were used in the following investigations.

To determine the effect of cytokines on the expression of miR‐27a‐3p, BMSCs from wild‐type mice were treated with the cytokines interleukin (IL)‐1β (1 ng/mL), interferon γ (IFN‐γ; 50 ng/mL) or TNF‐α (10 ng/mL) for 2 weeks, which was followed by determination of miR‐27a‐3p expression using RT–quantitative PCR (RT‐qPCR). During treatment, the culture medium was replaced every 2 days with fresh medium supplemented with the same concentrations of cytokines. No obvious toxic effects induced by these cytokines were identified.

### 2.5 RT‐qPCR

Total RNA was purified using TRIzol (AccuRef Scientific) and reverse‐transcribed into cDNA using a high‐capacity cDNA Reverse Transcription Kit (Thermo Fisher Scientific). qPCR was performed using SYBR Green master mix (Thermo Fisher Scientific). The housekeeping gene glyceraldehyde 3‐phosphate dehydrogenase (*GAPDH*) and *U6* were used as internal controls. The primers used in the study were as follows: miR‐27a‐3p, 5ʹ‐CGCGTTCACAGTGGCTAAGT‐3ʹ (forward) and 5ʹ‐GTGCAGGGTCCGAGGTATTC‐3ʹ (reverse); *Runx2*, 5ʹ‐CACCGACAGTCCCAACTTCCT‐3ʹ (forward), 5ʹ‐ACGGTAAC CA CAGTCCCATCTG‐3ʹ (reverse); *Osterix*, 5ʹ‐AGCGACCACTTGAGCAAACAT‐3ʹ (forward) and 5ʹ‐GCGGCTGATTGGCTTCTTCT‐3ʹ (reverse); *Sfrp1*, 5ʹ‐ACGTGGGCTACAAGAAGATGG‐3ʹ (forward) and 5ʹ‐CAGCGACACGGGTAGATGG‐3ʹ (reverse); *GAPDH*, 5ʹ‐CCATCACCATCTTCCAGGAGT‐3ʹ (forward) and 5ʹ‐GGATGATGTTCTGGAGAGCG‐3ʹ (reverse); *U6*, 5ʹ‐CTCGCTTCGGCAGCACA‐3ʹ (forward) and 5ʹ‐AACGCTTCACGAATTTGCGT‐3ʹ (reverse).

### 2.6 Western blot

Total protein was purified from BMSCs derived from the Sham or OVX mice, or cultured BMSCs using radioimmunoprecipitation assay (RIPA) buffer (AccuRef Scientific), and the protein concentration was determined using a BCA kit (Exprecision, Xi'an, China) according to the manufacturer's protocol. An equal amount of protein was separated using SDS‐PAGE and transferred onto a nitrocellulose membrane (Millipore, Billerica, MA, USA) for western blot analysis. The membranes were probed with the following primary antibodies: Runx2 (cat. no. 12556, Cell Signaling Technology, Danvers, MA, USA), Osterix (DF7731, Affinity Biosciences, Changzhou, China), Sfrp1 (cat. no. 3534, Cell Signaling Technology), β‐catenin (cat. no. 8480, Cell Signaling Technology), C‐Myc (cat. no. 18583, Cell Signaling Technology), Cyclin D1 (cat. no. 55506, Cell Signaling Technology), and the loading control, GAPDH (cat. no. 5174, Cell Signaling Technology). The membranes were further incubated with horseradish peroxidase‐conjugated secondary antibody (Cell Signaling Technology) and developed with an ECL kit (AccuRef Scientific).

### 2.7 Enzyme‐linked immunosorbent assay

Mouse serum samples from the Sham or OVX group were analysed by Mouse TNF‐α and IL‐1β enzyme‐linked immunosorbent assay (ELISA) kits (R&D Systems, Minneapolis, MN, USA) according to the manufacturer's instructions.

### 2.8 Haematoxylin and eosin staining

Mouse bone samples from the Sham or OVX group were fixed with 4% paraformaldehyde for 2 days and decalcified with 10% EDTA at room temperature for 4 weeks. Samples were then dehydrated with gradient ethanol, degreased in xylene, embedded in paraffin, and cut into the sequential 5 μm‐thick slices. The slices were then stained with haematoxylin and eosin (H&E) according to the manufacturer's instruction (Beyotime, China) to confirm the occurrence of osteoporosis in mice.

### 2.9 Luciferase reporter assay

Luciferase reporter vector containing wild type or a mutated version of 3ʹ‐UTR of *Sfrp1* was constructed using pGL3‐Luc vector (Promega, Madison, WI, USA). HEK293T cells were transfected with the indicated reporter vector, together with miR‐27a‐3p mimics or negative control. Relative luciferase activity was analysed with a Dual‐Luciferase reporter assay kit (Promega) 48 h later.

### 2.10 Alkaline phosphatase staining and Alizarin Red‐S staining

BMSCs from wild type mice were isolated and cultured in vitro for 14 days. Alkaline phosphatase (ALP) staining was performed to detect the differentiation of BMSCs using an alkaline phosphatase live stain kit (cat. no. ab242286, Abcam, Waltham, MA, USA) following the manufacturer's protocol. Differentiated BMSCs and control cells were also stained with aqueous 0.5% (v/v) Alizarin Red‐S (Sigma‐Aldrich, St Louis, MO, USA). The mineralized nodule formation was analysed and recorded under an inverted microscope (Olympus, Tokyo, Japan).

### 2.11 RNA immunoprecipitation assay

A RNA immunoprecipitation (RIP) assay was performed using the Dynabeads Protein G Immunoprecipitation Kit (Thermo Fisher Scientific). Briefly, dynabeads were incubated with anti‐Argonaute‐2 (AGO2) antibody (cat. no. ab186733, Abcam) or anti‐IgG antibody (cat. no. ab133470, Abcam) at room temperature for 30 min. Then, BMSC cell lysates were incubated with dynabeads‐anti‐AGO2 antibody mixture or dynabeads‐anti‐IgG antibody mixture at 4°C overnight. The next day, the RIP mixture was washed with RIP Wash Buffer, and proteinase K was used to digest the antibody. The pulled‐down RNA was purified by TRIzol for subsequent analysis.

### 2.12 Target prediction

The potential targets of miR‐27a‐3p were predicted by using microT (https://www.biostars.org/p/143874/) and miRDB (http://www.mirdb.org/mining.html). Subsequently, a Venn analysis was performed to screen the common targeted genes predicted by microT and miRDB.

### 2.13 Statistical analysis

All data obtained from the in vitro experiments (*n* = 3) or the in vivo experiments (*n* = 5) were presented as mean ± standard deviation. The statistical significance was analysed by two‐tailed Student's *t*‐test or one‐way analysis of variance (ANOVA) with post‐hoc Tukey's multiple comparisons test wherever necessary. GraphPad Prism v. 8.0 (GraphPad Software, Inc., San Diego, CA, USA) was used for statistical analysis. A *P* < 0.05 was considered statistically significant.

## 3 RESULTS

### 3.1 TNF‐α suppresses miR‐27a‐3p expression in OVX mice

**FIGURE 1:**
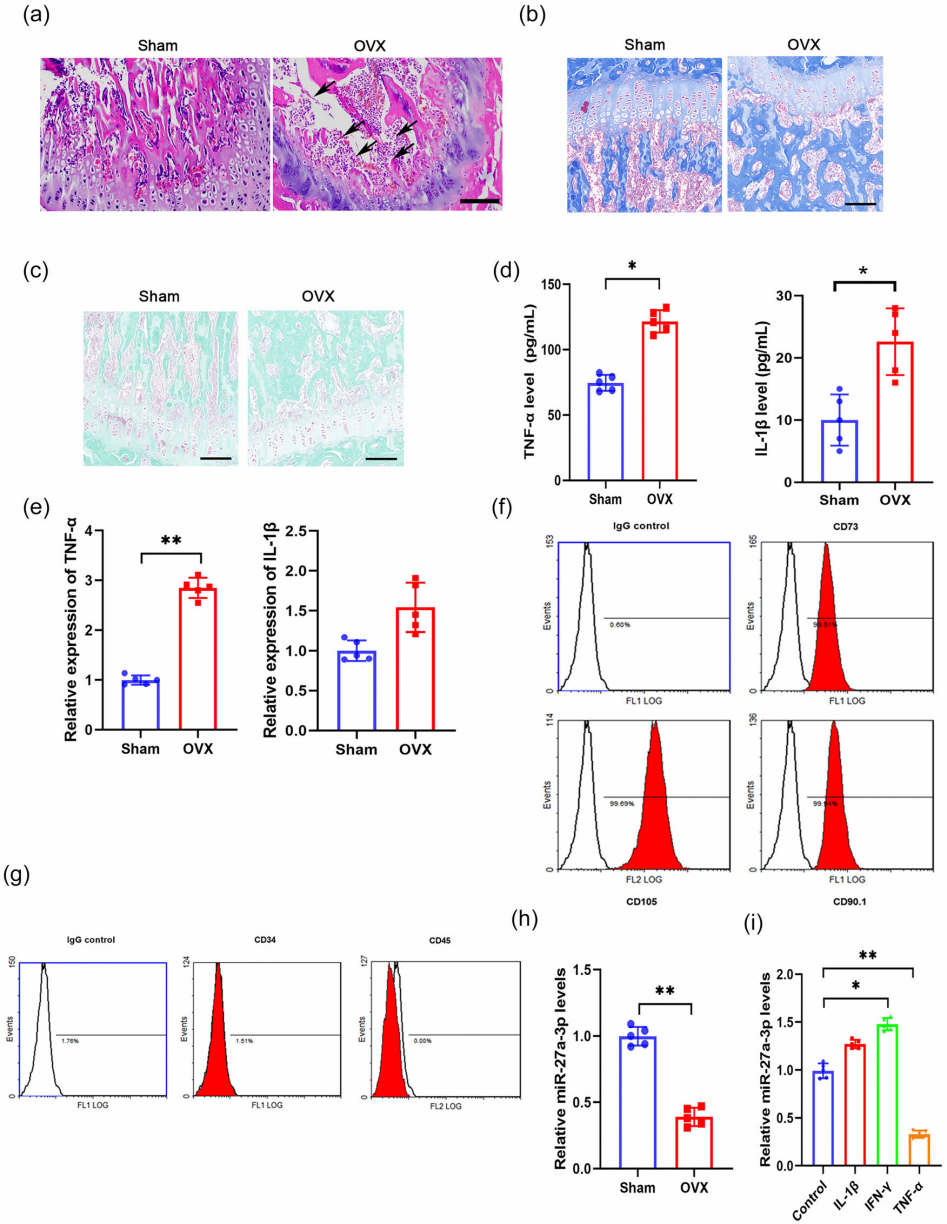
TNF‐α suppresses miR‐27a‐3p expression in ovariectomized (OVX) mice. (a–e) Wild‐type (WT) C57BL/6 mice underwent bilateral ovariectomy surgeries to establish the OVX mouse model, and mice that underwent sham operation were used as controls. (a–c) Representative haematoxylin and eosin (H&E) staining (a), Masson staining (b) and Goldner staining (c) of bone tissues from the Sham or OVX group to confirm osteoporosis (arrow indicates osteoporosis). Scale bar, 100 μm. (d) The tumour necrosis factor α (TNF‐α) and interleukin (IL)‐1β levels in the serum were analysed by ELISA at 60 days after modelling. (e) The relative mRNA expression levels of TNF‐α and IL‐1β in bone tissue were analysed by qPCR. (f, g) Representative flow cytometrical histogram plots show that bone marrow mesenchymal cells (BMSCs) isolated from mice bones were positive for BMSC markers CD73, CD105 and CD90.1 (f), and were negative for haematopoietic markers CD34 and CD45 (g). (h) The relative expression of miR‐27a‐3p in BMSCs from the Sham or OVX group was analysed by qPCR. (i) BMSCs from WT mice were treated with IL‐1β (1 ng/mL), interferon γ (IFN‐γ; 50 ng/mL), or TNF‐α (10 ng/mL) for 2 weeks, and the relative expression of miR‐27a‐3p was analysed by qPCR. *P < 0.05, **P < 0.01, between the indicated groups.

Previous studies have demonstrated that TNF‐α plays a critical role in osteogenesis (Yang et al., 2013). To confirm the function and explore the underlying mechanism of TNF‐α in osteogenesis, we examined the expression level of TNF‐α in the OVX animal model. Wild type mice were subjected to either a sham operation or a bilateral ovariectomy to establish the OVX mouse model. Osteoporosis in OVX mice was confirmed by H&E (Figure 1a), Masson (Figure 1b) and Goldner staining (Figure 1c). The TNF‐α and IL‐1β levels in OVX mouse serum were significantly higher than those in the Sham group (Figure 1d; *P* = 0.0106 and 0.0147 for TNF‐α and IL‐1β, respectively; *n* = 5 for each group). Significantly increased expression of TNF‐α mRNA was also confirmed in the bone tissues of the OVX mice (Figure 1e; *P* = 0.003; *n* = 5 for each group). Although an obvious upregulation of IL‐1β mRNA was observed in the OVX group, no significant difference was identified between the OVX and Sham group (Figure 1e; *P* = 0.07; *n* = 5 for each group). To understand the functional role of TNF‐α, we performed next‐generation sequencing using the total RNA from BMSCs isolated from the bone marrow of the Sham and OVX mice and found that multiple miRNAs were markedly dysregulated in expression between the Sham and OVX group (data not shown). MiR‐27a‐3p, which regulates cell proliferation in various tumours (Chen et al., 2021; Su et al., 2019; Yang et al., 2021), was found significantly downregulated in OVX mice. To further explore the regulatory relationship between cytokines and miR‐27a‐3p, BMSCs were isolated and characterized by their surface markers using flow cytometry (Figure 1f,g). The downregulation of miR‐27a‐3p in BMSCs from OVX mice was further confirmed by qPCR (Figure 1h; *P* = 0.003; *n* = 5 for each group). BMSCs from the Sham group were treated with different inflammatory cytokines (IL‐1β, IFN‐γ or TNF‐α) to verify the regulation of miR‐27a‐3p. As shown in Figure 1i, TNF‐α treatment significantly downregulated the expression of miR‐27a‐3p (*P* = 0.0032), while IFN‐γ markedly increased the expression of miR‐27a‐3p (*P* = 0.0188). However, IL‐1β did not produce any discernible effects on the expression of miR‐27a‐3p (*P* = 0.5148; *n* = 5 for each group).

### 3.2 MiR‐27a‐3p promotes osteogenic differentiation of BMSCs in vitro

**FIGURE 2:**
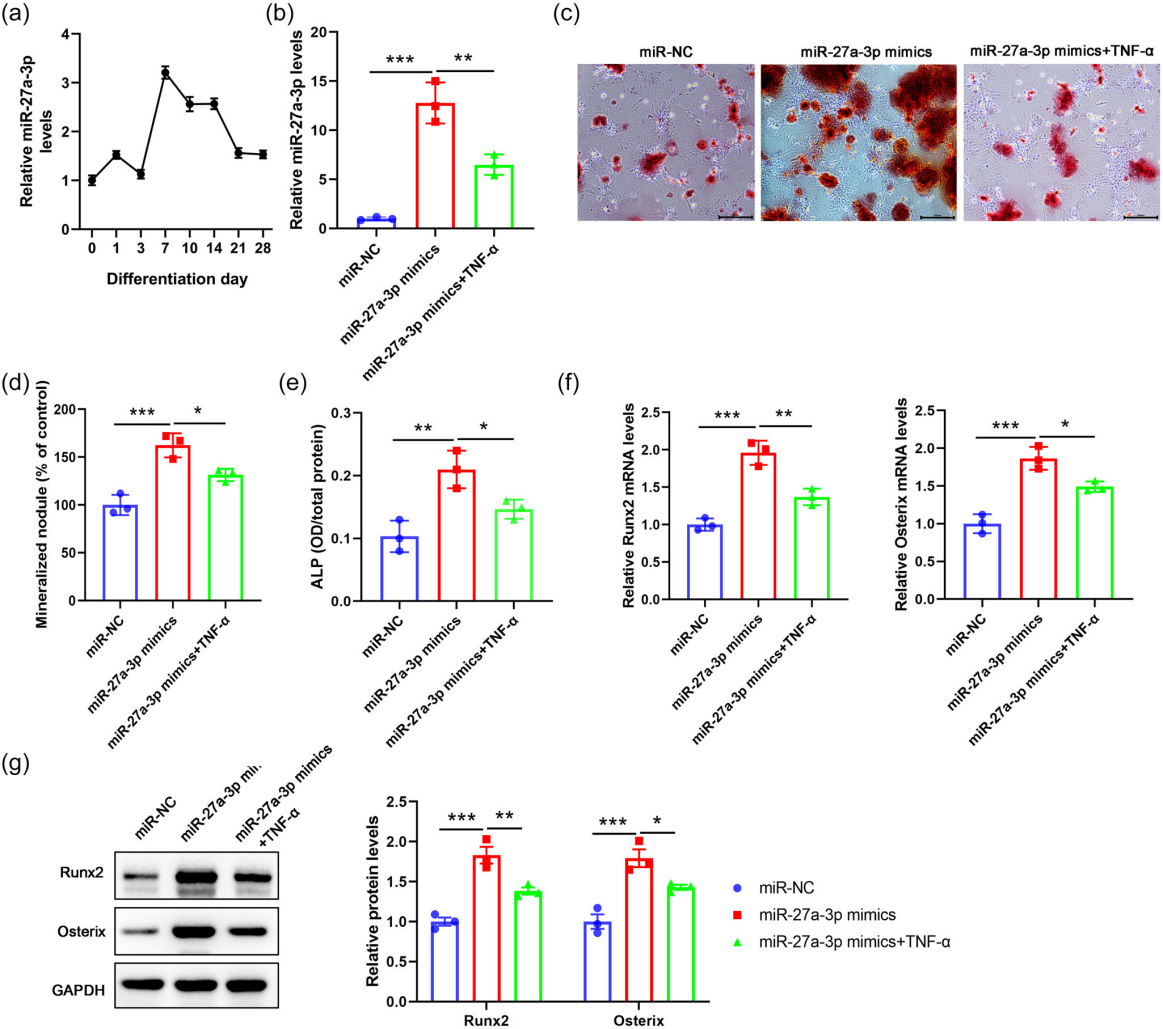
MiR‐27a‐3p promotes osteogenic differentiation of BMSCs in vitro. (a–g) BMSCs were isolated from WT mice and cultured in vitro. (a) The expression of miR‐27a‐3p was analysed by qPCR at the indicated time points. (b) BMSCs were transfected with miR‐NC, miR‐27a‐3p mimics followed by TNF‐α treatment (10 ng/mL). The expression of miR‐27a‐3p was analysed by qPCR 48 h later. (c) Transfected BMSCs were cultured in vitro for 7 days, and osteogenic differentiation of BMSCs was subsequently analysed by Alizarin Red staining. Scale bar, 100 μm. (d) Quantification result for Alizarin Red staining. (e) The expression of ALP was analysed by ALP staining. (f) Transfected BMSCs were cultured in vitro for 7 days and the mRNA expression of Runx2 and Osterix was analysed by qPCR. (g) Transfected BMSCs were cultured in vitro for 7 days and the protein expression of Runx2 and Osterix was analysed by western blot. *P < 0.05, **P < 0.01, ***P < 0.001, between the indicated groups.

To further characterize the function of miR‐27a‐3p in osteogenesis, BMSCs were isolated from wild type mice and cultured in vitro for osteogenic differentiation. The expression of miR‐27a‐3p was analysed at different time points (Figure 2a). We found that miR‐27a‐3p expression reached the peak level at around day 7 and decreased gradually, thus indicating a promotive role of miR‐27a‐3p in osteogenic differentiation (Figure 2a).

Following this observation, we transfected BMSCs with miR‐27a‐3p mimics and treated them with TNF‐α for further analyses. We confirmed that additional TNF‐α stimulation downregulated miR‐27a‐3p expression (Figure 2b; *P* < 0.001, miR‐NC vs. miR‐27a‐3p mimics; *P* = 0.003, miR‐27a‐3p mimics vs. miR‐27a‐3p mimics+TNF‐α; *n* = 3 for each group). In addition, overexpression of miR‐27a‐3p significantly enhanced osteoblast differentiation, as evidenced by increased development of mineralized nodules and upregulated ALP expression, while TNF‐α treatment markedly aborted this enhancement of osteogenic differentiation induced by miR‐27a‐3p mimics (Figure 2c–e; for mineralized nodule, *P* < 0.001, miR‐NC vs. miR‐27a‐3p mimics; *P* = 0.02, miR‐27a‐3p mimics vs. miR‐27a‐3p mimics+TNF‐α; for ALP, *P* = 0.004, miR‐NC vs. miR‐27a‐3p mimics; *P* = 0.04, miR‐27a‐3p mimics vs. miR‐27a‐3p mimics+TNF‐α; *n* = 3 for each group). Moreover, we found that the overexpression of miR‐27a‐3p elevated the mRNA and protein levels of osteoblast‐specific markers Runx2 and Osterix (Figure 2f,g; miR‐NC vs. miR‐27a‐3p mimics, for *Runx2* mRNA, *P* < 0.001; for *Osterix* mRNA, *P* < 0.001; for Runx2 protein, *P* < 0.001; for Osterix protein, *P* < 0.001; *n* = 3 for each group). As expected, additional TNF‐α treatment aborted the upregulation in expression of Runx2 and Osterix (Figure 2f,g; miR‐NC vs. miR‐27a‐3p mimics, for *Runx2* mRNA, *P* = 0.003; for *Osterix* mRNA, *P* = 0.02; for Runx2 protein, *P* = 0.005; for Osterix protein, *P* = 0.02; *n* = 3 for each group). Collectively, these data suggested that miR‐27a‐3p enhanced osteogenic differentiation of BMSCs in vitro and TNF‐α treatment could attenuate this progression via inhibiting miR‐27a‐3p.

### 3.3 MiR‐27a‐3p negatively regulates Sfrp1 expression by binding to the 3ʹ‐UTR of its mRNA

**FIGURE 3:**
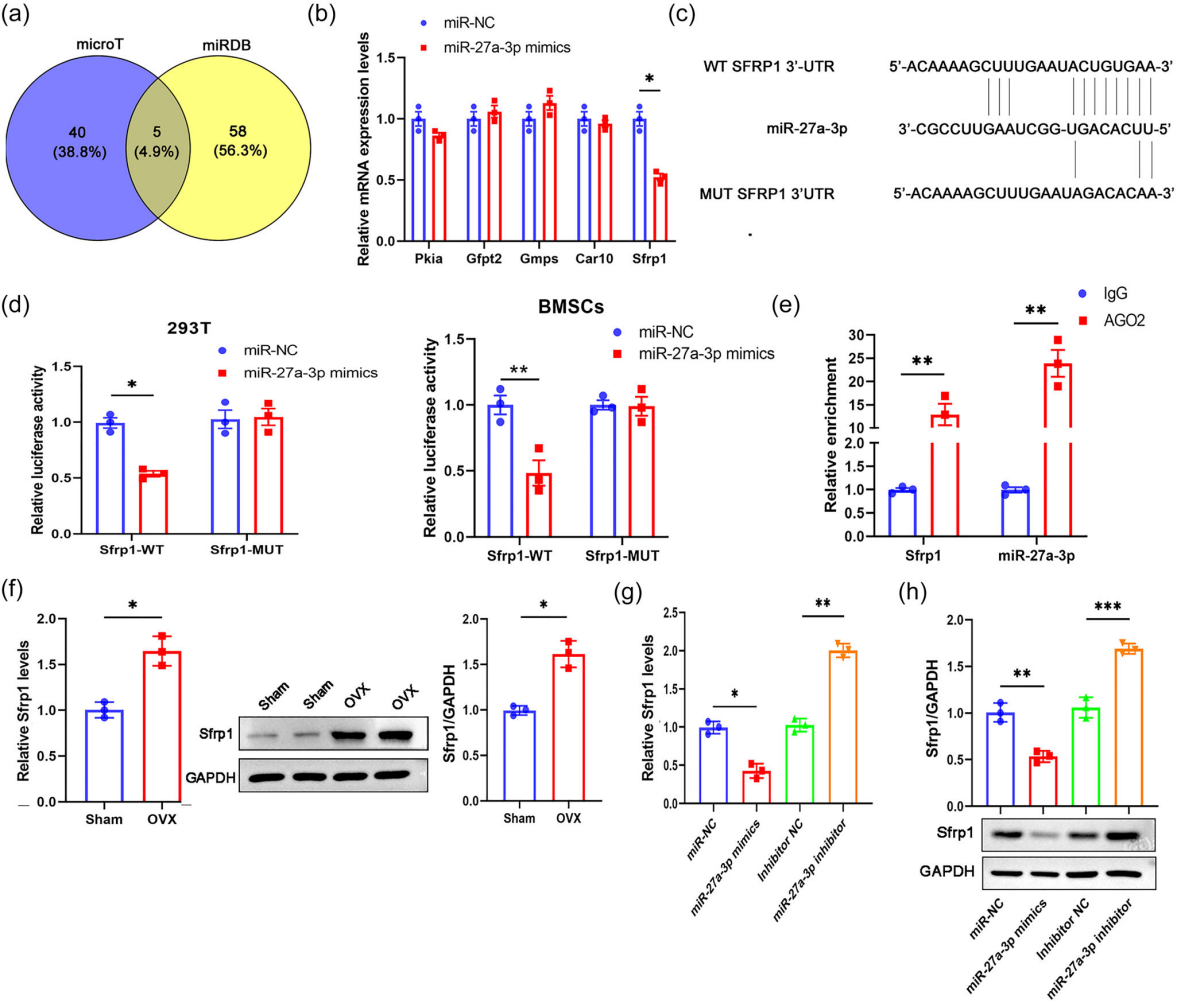
Sfrp1 is a direct target of miR‐27a‐3p. (a) Bioinformatics analysis was performed using the online tools microT and miRDB to predict the potential targets of miR‐27a‐3p. (b) BMSCs from wild‐type (WT) mice were transfected with miR‐NC or miR‐27a‐3p mimics. The relative expressions of potential target genes (*Pkia*, *Gfpt2*, *Gmps*, *Car10* and *Sfrp1*) were analysed by qPCR. (c) The complementary binding sequences between miR‐27a‐3p and WT or mutated (MUT) 3ʹ‐UTR of *Sfrp1* mRNA are shown. (d) HEK293T cells and BMSCs were transfected with miR‐NC or miR‐27a‐3p mimics, together with luciferase reporter vector containing WT or Mut 3ʹ‐UTR of *Sfrp1* mRNA. The relative luciferase activity was analysed 48 h later. (e) BMSCs were isolated from wild‐type mice and RNA immunoprecipitation assay was performed using an isotype control IgG or anti‐AGO2 antibody. The relative enrichment of *Sfrp1* and miR‐27a‐3p was analysed by qPCR. (f) BMSCs were isolated from the Sham or OVX mice. The mRNA and protein expression of Sfrp1 was analysed by qPCR or western blot. (g, h) BMSCs isolated from WT mice were transfected with miR‐NC, miR‐27a‐3p mimics, inhibitor NC, or miR‐27a‐3p inhibitor. The expression of Sfrp1 mRNA (g) or protein (h) 7 days later was analysed by qPCR or western blot assays, respectively. *P < 0.05, **P < 0.011, ***P < 0.001, between the indicated groups.

To further reveal the molecular mechanisms under which TNF‐α regulates miR‐27a‐3p expression in BMSCs, we next predicted the downstream targets of miR‐27a‐3p with online tools. In total, 45 genes were predicted by microT and 63 genes were predicted by miRDB, with five common targets predicted by both online tools (Figure 3a, Table S1). Thus, five genes (*Pkia*, *Gfpt2*, *Gmps*, *Car10* and *Sfrp1*) were considered as the potential targets of miR‐27a‐3p, and the relative expression of these genes in BMSCs transfected with miR‐NC or miR‐27a‐3p mimics was examined by qPCR. As shown in Figure 3b (miR‐NC vs. miR‐27a‐3p mimic, *P* = 0.14 for *Pkia*; *P* = 0.21 for *Gfpt*; *P* = 0.1611 for *Gmps*; *P* = 0.2623 for *Car10*; *P* = 0.04 for *Sfrp1*; *n* = 3 for each group), *Sfrp1* was the only gene whose mRNA expression was significantly inhibited by miR‐27a‐3p mimics. Further analysis indicated that miR‐27a‐3p had complementary binding sequences that targeted the 3ʹ‐UTR of wild type *Sfrp1* mRNA (Figure 3c). The luciferase reporter assay confirmed that miR‐27a‐3p mimics suppressed the relative luciferase activity in HEK293T cells and BMSCs transfected with luciferase reporter containing the wild type (WT) 3ʹ‐UTR of *Sfrp1* mRNA, but not with the mutated 3ʹ‐UTR of *Sfrp1* mRNA (Figure 3d; miR‐NC vs. miR‐27a‐3p mimic, in 293T cells, *P* = 0.032 for *Sfrp1* WT; *P* = 0.5612 for *Sfrp1* MUT; in BMSCs, *P* = 0.002 for *Sfrp1* WT; *P* = 0.9943 for *Sfrp1* MUT; *n* = 3 for each group). In addition, the interaction between *Sfrp1* and miR‐27a‐3p was validated by the RIP assay in BMSCs. Anti‐AGO2 immunoprecipitation was observed to markedly enhance the enrichment of *Sfrp1* and miR‐27a‐3p, compared to the immunoprecipitation with the IgG control, suggesting that *Sfrp1* could directly interact with miR‐27a‐3p (Figure 3e; IgG vs. AGO2, *P* = 0.004 for *Sfrp1*; *P* = 0.0022 for miR‐27a‐3p mimic; *n* = 3 for each group). Additionally, BMSCs from OVX mice had significantly more Sfrp1 expression at both mRNA and protein levels than that from the mice in the Sham group (Figure 3f; Sham vs. OVX, *P* = 0.0256 for *Sfrp1* mRNA; *P* = 0.0287 for Sfrp1 protein; *n* = 3 for each group). Moreover, we confirmed that miR‐27a‐3p overexpression inhibited the expression of Sfrp1 in BMSCs, while inhibition of miR‐27a‐3p elevated the expression of Sfrp1 (Figure 3g,h; for *Sfrp1* mRNA, *P* = 0.0242, miR‐NC vs. miR‐27a‐3p mimic; *P* = 0.0059, Inhibitor NC vs. miR‐27a‐3p inhibitor; for Sfrp1 protein, *P* = 0.0023, miR‐NC vs. miR‐27a‐3p mimic; *P* < 0.001, Inhibitor NC vs. miR‐27a‐3p inhibitor; *n* = 3 for each group). Taken together, Sfrp1 was concluded to be a direct target of miR‐27a‐3p in BMSCs during osteogenic differentiation.

### 3.4 Overexpression of *Sfrp1* inhibits osteogenic differentiation of BMSCs in vitro

**FIGURE 4:**
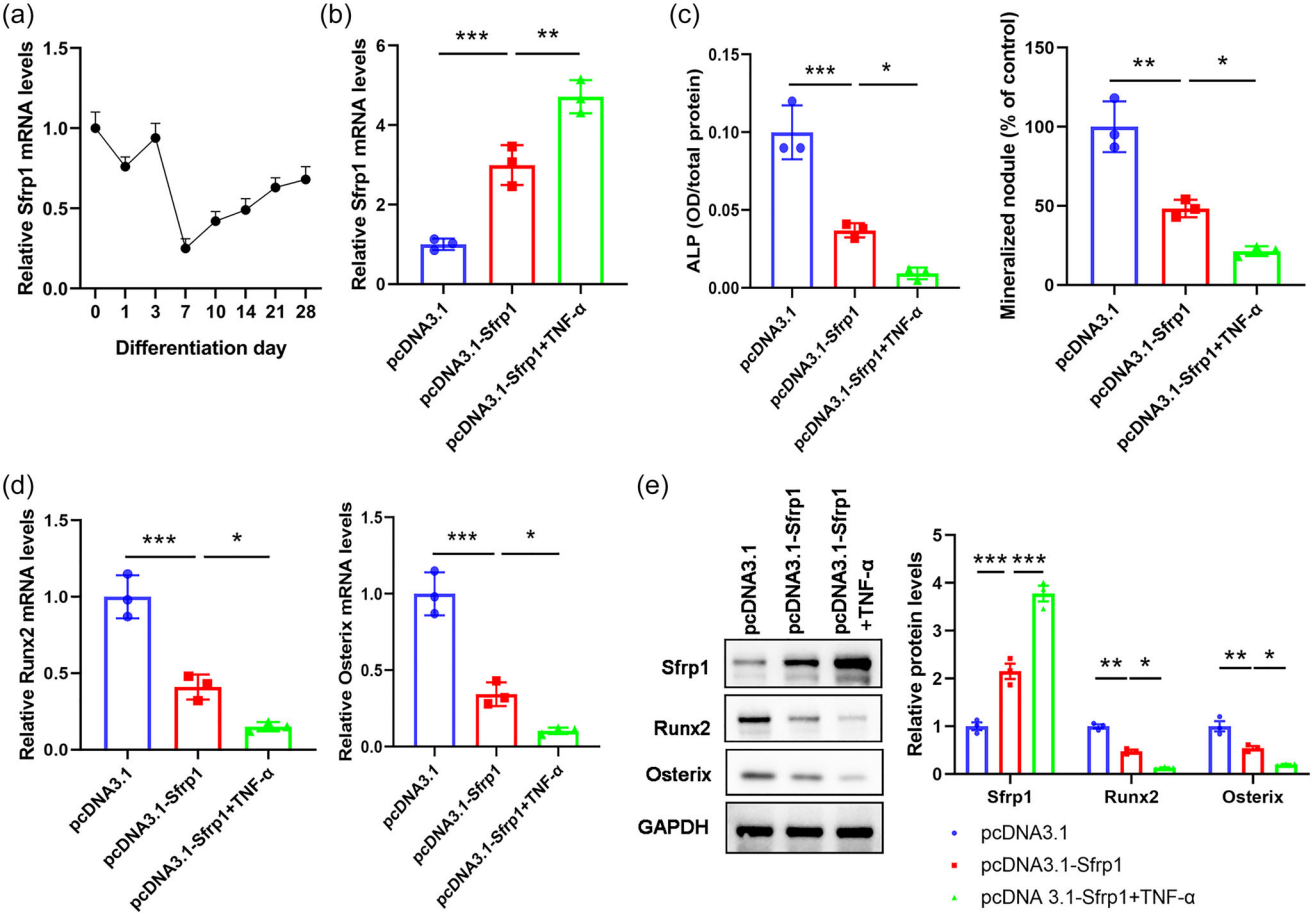
Knockdown of *Sfrp1* promotes osteogenic differentiation of BMSCs in vitro. (a) BMSCs were isolated from wild‐type (WT) mice and cultured in vitro. The expression of *Sfrp1* was analysed by qPCR at the indicated time points. (b–e) BMSCs were transfected with empty vector pcDNA3.1, or *Sfp1*‐expressing vector pcDNA3.1‐Sfrp1 alone or followed by TNF‐α treatment (10 ng/mL). (b) The expression of *Sfrp1* was analysed by qPCR 48 h later. (c–e) Transfected BMSCs were cultured in vitro for 7 days. (c) The expression of ALP and formation of mineralized nodule were subsequently analysed by ALP staining and Alizarin Red‐S staining, respectively. (d) The mRNA expression of *Runx2* and *Osterix* was analysed by qPCR. (e) The protein expression of Runx2 and Osterix was analysed by western blot. *P < 0.05, **P < 0.01, ***P < 0.001, between the indicated groups.

To further characterize the functional role of Sfrp1, we measured the expression level of *Sfrp1* at different time points during osteogenic differentiation. In contrast to the expression of miR‐27a‐3p, which reached the peak level at day 7, *Sfrp1* expression gradually decreased and reached the lowest level at day 7 (Figure 4a). We then overexpressed *Sfrp1* in BMSCs, which was followed by TNF‐α treatment (Figure 4b; *P* < 0.001, pcDNA3.1 vs. pcDNA3.1‐Sfrp1; *P* = 0.004, pcDNA3.1‐Sfrp1 vs. pcDNA3.1‐Sfrp1+TNF‐α; *n* = 3 for each group). Consistently, we revealed that overexpression of *Sfrp1* significantly decreased osteoblast differentiation, as shown as decreased mineralized nodules and downregulated ALP expression, while additional TNF‐α treatment further enhanced this inhibition (Figure 4c; pcDNA3.1 vs. pcDNA3.1‐Sfrp1, *P* < 0.001 for ALP, *P* = 0.002 for mineralized nodule; pcDNA3.1‐Sfrp1 vs. pcDNA3.1‐Sfrp1+TNF‐α, *P* = 0.04 for ALP, *P* = 0.04 for mineralized nodule; *n* = 3 for each group). Furthermore, we found that overexpression of *Sfrp1* also obviously decreased the expression of Runx2 and Osterix (Figure 4d,e; pcDNA3.1 vs. pcDNA3.1‐Sfrp1, *P* < 0.001 for *Runx2* mRNA, *P* < 0.001 for *Osterix* mRNA, *P* < 0.001 for Sfrp1 protein, *P* = 0.002 for Runx2 protein, *P* = 0.007 for Osterix protein; pcDNA3.1‐Sfrp1 vs. pcDNA3.1‐Sfrp1+TNF‐α, *P* = 0.04 for *Runx2* mRNA, *P* = 0.05 for *Osterix* mRNA, *P* < 0.001 for Sfrp1 protein, *P* = 0.04 for Runx2 protein, *P* = 0.05 for Osterix protein; *n* = 3 for each group). These results suggested that TNF‐α treatment could attenuate osteogenic differentiation of BMSCs in vitro via upregulating Sfrp1 expression.

### 3.5 TNF‐α treatment upregulates *Sfrp1* expression to abrogate the promotive effect of miR‐27a‐3p on osteogenic differentiation of BMSCs

**FIGURE 5:**
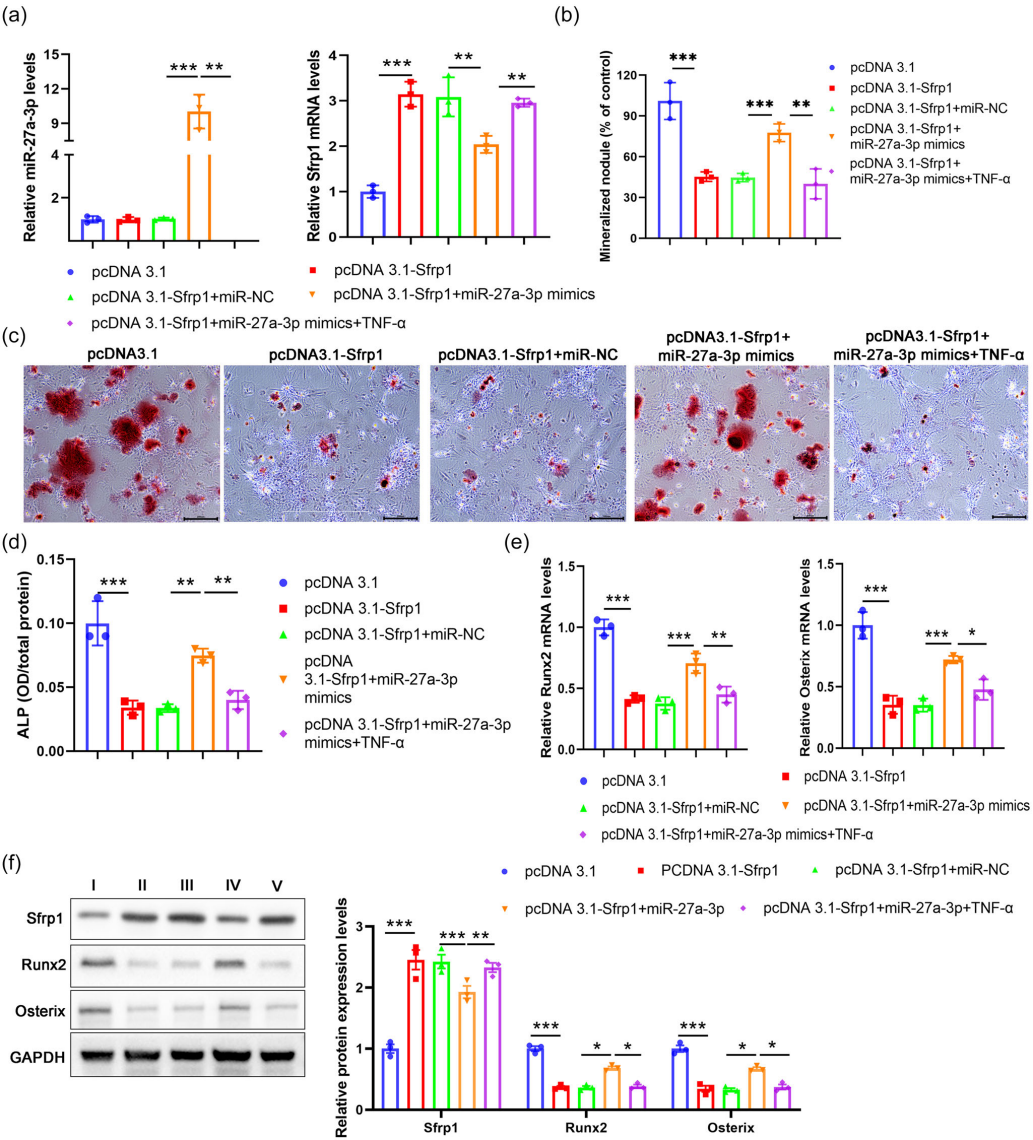
Overexpression of Sfrp1 abrogates the promotive effect of miR‐27a‐3p on osteogenic differentiation of BMSCs. (a–f) BMSCs from wild‐type (WT) mice were transfected with pcDNA 3.1, or pcDNA3.1‐Sfrp1, or (pcDNA3.1‐Sfrp1+miR‐NC), or (pcDNA3.1‐Sfrp1+miR‐27a‐3p mimics) alone or followed by TNF‐α treatment (10 ng/mL). (a) The relative expression of miR‐27a‐3p and *Sfrp1* was examined by qPCR 48 h later. (b–f) Transfected cells were cultured for 7 days. (b, c) Formation of mineralized nodule was analysed by Alizarin Red‐S staining. Scale bar, 100 μm. (d) The expression of ALP was analysed by ALP staining. (e) The mRNA expression of *Runx2* and *Osterix* was analysed by qPCR. (f) The protein expression of Sfrp1, Runx2 and Osterix was analysed by western blot. *P < 0.05, **P < 0.01, ***P < 0.001, between the indicated groups.

We performed rescue experiments to validate the interactions between miR‐27a‐3p and *Sfrp1* in osteogenic differentiation. BMSCs were transfected with empty vector (pcDNA3.1), or *Sfp1*‐expressing vector (pcDNA3.1‐Sfrp1), or empty vector and control miRNA (pcDNA3.1‐Sfrp1 + miR‐NC), or *Sfp1*‐expressing vector and miR‐27a‐3p miRNA (pcDNA3.1‐Sfrp1 + miR‐27a‐3p mimics), or *Sfp1*‐expressing vector and miR‐27a‐3p miRNA along with additional TNF‐α treatment (pcDNA3.1‐Sfrp1 + miR‐27a‐3p mimics + TNF‐α), followed by osteogenic differentiation determination. The results showed that overexpression of *Sfrp1* significantly increased *Sfrp1* expression in BMSCs but had no effect on miR‐27a‐3p expression (Figure 5a; for miR‐27a‐3p expression, *P* > 0.99, pcDNA3.1 vs. pcDNA3.1‐Sfrp1; *P* < 0.001, pcDNA 3.1‐Sfrp1+miR‐NC vs. pcDNA 3.1‐Sfrp1+miR‐27a‐3p mimics; *P* = 0.002, pcDNA 3.1‐Sfrp1+miR‐27a‐3p mimics vs. pcDNA 3.1‐Sfrp1+miR‐27a‐3p mimics+TNF‐α; *n* = 3 for each group). However, overexpression of miR‐27a‐3p mimic markedly increased miR‐27a‐3p expression but suppressed the *Sfrp1* expression in BMSCs (Figure 5a; for *Sfrp1* expression, *P* < 0.001, pcDNA3.1 vs. pcDNA3.1‐Sfrp1; *P* = 0.004, pcDNA 3.1‐Sfrp1+miR‐NC vs. pcDNA 3.1‐Sfrp1+miR‐27a‐3p mimics; *P* = 0.009, pcDNA 3.1‐Sfrp1+miR‐27a‐3p mimics vs. pcDNA 3.1‐Sfrp1+miR‐27a‐3p mimics+TNF‐α; *n* = 3 for each group). Further analyses showed that transfection of pcDNA3.1‐Sfrp1 plasmid markedly inhibited osteogenic differentiation of BMSCs, as evidenced by the decreases in mineralized nodule development and ALP expression in comparison to the control group, while overexpression of miR‐27a‐3p markedly reversed this inhibition (Figure 5b–d; pcDNA3.1 vs. pcDNA3.1‐Sfrp1, *P* < 0.001 for mineralized nodule, *P* < 0.001 for ALP expression; pcDNA 3.1‐Sfrp1+miR‐NC vs. pcDNA 3.1‐Sfrp1+miR‐27a‐3p mimics, *P* = 0.009 for mineralized nodule, *P* = 0.002 for ALP expression; pcDNA 3.1‐Sfrp1+miR‐27a‐3p mimics vs. pcDNA 3.1‐Sfrp1+miR‐27a‐3p mimics+TNF‐α, *P* = 0.005 for mineralized nodule, *P* = 0.005 for ALP expression; *n* = 3 for each group). However, TNF‐α treatment obviously aborted the effects of miR‐27a‐3p in promoting osteogenic differentiation of *Sfrp1*‐overexpressed BMSCs, as shown as the decreases in mineralized nodule development and ALP expression (Figure 5b–d). Consistently, overexpression of *Sfrp1* markedly decreased Runx2 and Osterix expression in BMSCs, but overexpression of miR‐27a‐3p significantly antagonized the inhibitive effect of Sfrp1 on Runx2 and Osterix expression (Figure 5e,f). In addition, TNF‐α treatment obviously aborted the effects of miR‐27a‐3p in elevating the expression levels of Runx2 and Osterix in *Sfrp1*‐overexpressed BMSCs (Figure 5e,f; pcDNA3.1 vs. pcDNA3.1‐Sfrp1, *P* < 0.001 for *Runx2* mRNA, *P* < 0.001 for *Osterix* mRNA, *P* < 0.001 for Sfrp1 protein, *P* < 0.001 for Runx2 protein, *P* < 0.001 for Osterix protein; pcDNA 3.1‐Sfrp1+miR‐NC vs. pcDNA 3.1‐Sfrp1+miR‐27a‐3p mimics, *P* < 0.001 for *Runx2* mRNA, *P* < 0.001 for *Osterix* mRNA, *P* < 0.001 for Sfrp1 protein, *P* = 0.03 for Runx2 protein, *P* = 0.01 for Osterix protein; pcDNA 3.1‐Sfrp1+miR‐27a‐3p mimics vs. pcDNA 3.1‐Sfrp1+miR‐27a‐3p mimics+TNF‐α, *P* = 0.003 for *Runx2* mRNA, *P* = 0.02 for *Osterix* mRNA, *P* = 0.003 for Sfrp1 protein, *P* = 0.04 for Runx2 protein, *P* = 0.05 for Osterix protein; *n* = 3 for each group). Thus, our findings suggested that TNF‐α regulated the osteogenic differentiation of BMSCs via negatively regulating the miR‐27a‐3p–Sfrp1 axis.

### 3.6 The miR‐27a‐3p–Sfrp1 axis regulates β‐catenin–c‐Myc–Cyclin D1 signalling in osteogenic differentiation of BMSCs

**FIGURE 6:**
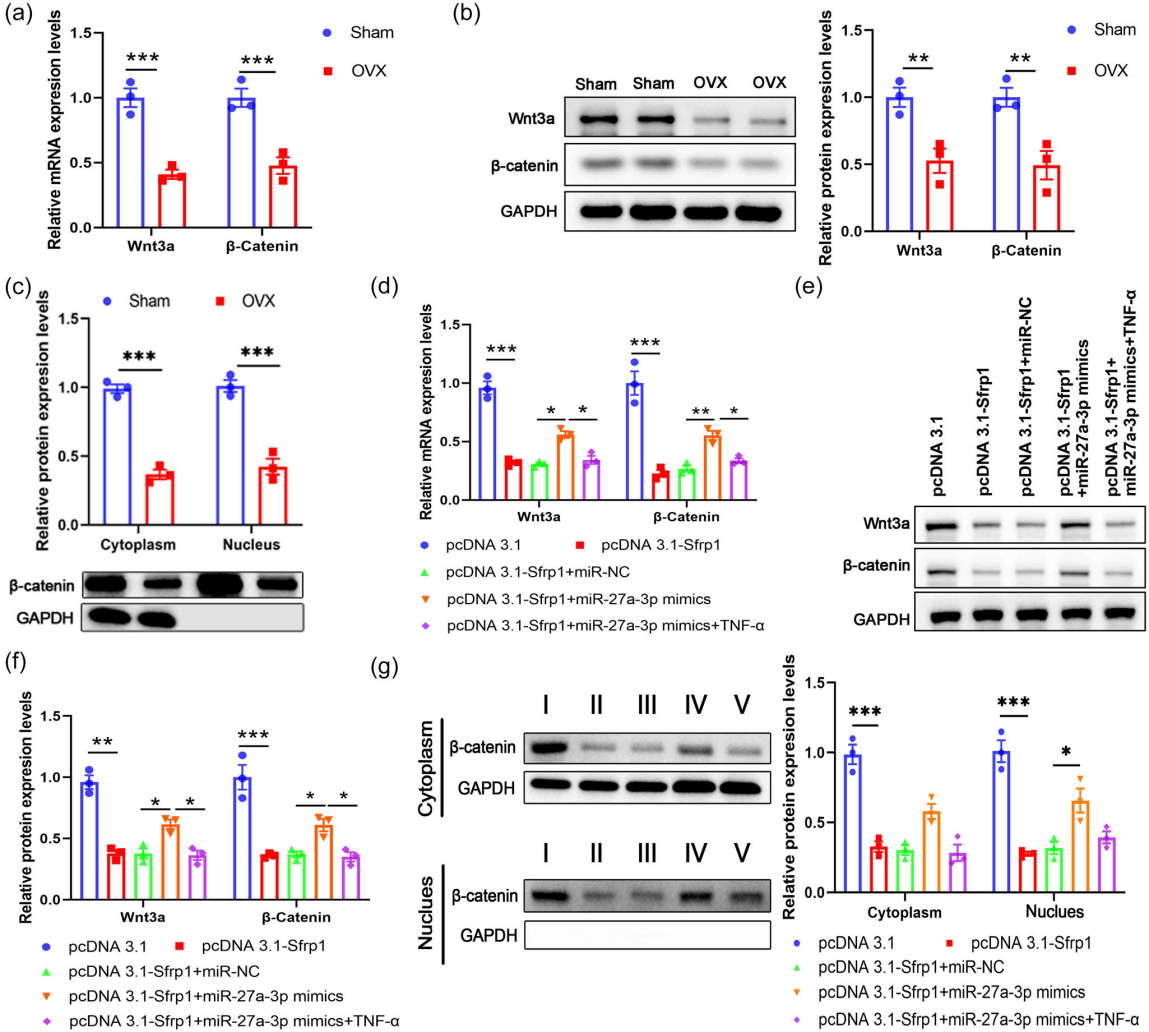
MiR‐27a‐3p–Sfrp1 axis regulates the Wnt3a–β‐catenin signalling in osteogenic differentiation of BMSCs. (a–c) BMSCs from the Sham or OVX mice were isolated. The mRNA (a) and protein (b) expression of Wnt3a and β‐catenin were analysed by qPCR and western blot, respectively. (c) The expression levels of β‐catenin in nucleus and cytoplasm were determined by western blot. (d–g) BMSCs from wild‐type mice were transfected with pcDNA3.1, or pcDNA3.1‐Sfrp1, or (pcDNA3.1‐Sfrp1 + miR‐NC), or (pcDNA3.1‐Sfrp1 + miR‐27a‐3p mimics) alone or followed by TNF‐α treatment (10 ng/mL). The mRNA (d) and protein (e, f) expression of Wnt3a and β‐catenin were analysed by qPCR and western blot, respectively. (g) The expression levels of β‐catenin in nucleus and cytoplasm were determined by western blot. *P < 0.05, **P < 0.01, ***P < 0.001, between the indicated groups.

Increasing evidence has indicated that the Wnt–β‐catenin signalling is involved in osteogenic differentiation (Baron & Gori, 2018; Manolagas, 2014). Therefore, we analysed the expression levels of Wnt3a and β‐catenin, two key molecules of Wnt–β‐catenin signalling, in BMSCs from the Sham or OVX mice. We found that the Wnt–β‐catenin signalling was inhibited in BMSCs from OVX mice, as evidenced by reduced expression of both Wnt3a and β‐catenin at both mRNA and protein levels (Figure 6a,b; Sham vs. OVX, *P* < 0.001 for *Wnt3a* mRNA, *P* < 0.001 for *β‐catenin* mRNA, *P* = 0.009 for Wnt3a protein, *P* = 0.006 for β‐catenin protein; *n* = 3 for each group). In addition, we also detected the level of β‐catenin nuclear translocation. Compared to the Sham group, the OVX group demonstrated significantly decreased expression level of β‐catenin in nucleus or cytoplasm (Figure 6c; Sham vs. OVX, *P* = 0.0001 for Cytoplasm, *P* = 0.0003 for Nucleus; *n* = 3 for each group). To test whether the miR‐27a‐3p–Sfrp1 axis regulates Wnt–β‐catenin signalling, BMSCs were transfected with pcDNA3.1, or pcDNA3.1‐Sfrp1, or (pcDNA3.1‐Sfrp1 + miR‐NC), or (pcDNA3.1‐Sfrp1 + miR‐27a‐3p mimics) alone or followed by TNF‐α treatment. *Sfrp1* overexpression suppressed Wnt–β‐catenin signalling, while miR‐27a‐3p mimics aborted the effects of overexpressed *Sfrp1* effects in elevating the expression levels of Wnt3a and β‐catenin (Figure 6d–f). Moreover, additional TNF‐α treatment attenuated the influence of miR‐27a‐3p on the Wnt–β‐catenin signalling, as shown as the increase in the expression of Wnt3a and β‐catenin in BMSCs transfected with *Sfrp1*‐expressing plasmid (Figure 6d–f; pcDNA3.1 vs. pcDNA3.1‐Sfrp1, *P* < 0.001 for *Wnt3a* mRNA, *P* < 0.001 for *β‐catenin* mRNA, *P* < 0.001 for Wnt3a protein, *P* < 0.001 for β‐catenin protein; pcDNA 3.1‐Sfrp1+miR‐NC vs. pcDNA 3.1‐Sfrp1+miR‐27a‐3p mimics, *P* = 0.01 for *Wnt3a* mRNA, *P* = 0.003 for *β‐catenin* mRNA, *P* = 0.04 for Wnt3a protein, *P* = 0.04 for β‐catenin protein; pcDNA 3.1‐Sfrp1+miR‐27a‐3p mimics vs. pcDNA 3.1‐Sfrp1+miR‐27a‐3p mimics+TNF‐α, *P* = 0.05 for *Wnt3a* mRNA, *P* = 0.05 for *β‐catenin* mRNA, *P* = 0.03 for Wnt3a protein, *P* = 0.02 for β‐catenin protein; *n* = 3 for each group). Furthermore, the changes in the expression level of β‐catenin in nucleus or cytoplasm among the tested groups were consistent with that observed for the total protein level of β‐catenin (Figure 6g; pcDNA3.1 vs. pcDNA3.1‐Sfrp1, *P* < 0.001 for Cytoplasmic β‐catenin, *P* < 0.001 for Nuclear β‐catenin; pcDNA 3.1‐Sfrp1+miR‐NC vs. pcDNA 3.1‐Sfrp1+miR‐27a‐3p mimics, *P* = 0.13 for Cytoplasmic β‐catenin, *P* = 0.01 for Nuclear β‐catenin; pcDNA 3.1‐Sfrp1+miR‐27a‐3p mimics vs. pcDNA 3.1‐Sfrp1+miR‐27a‐3p mimics+TNF‐α, *P* = 0.2 for Cytoplasmic β‐catenin, *P* = 0.07 for Nuclear β‐catenin; *n* = 3 for each group). In summary, TNF‐α modulated the osteogenic differentiation of BMSCs by inhibiting the miR‐27a‐3p–Sfrp1 axis and regulating Wnt–β‐catenin signalling.

## 4 DISCUSSION

The imbalance between osteoblastic bone formation and resorption leads to osteoporosis, which is a common disease among elderly people (Boyle et al., 2003; Teitelbaum, 2000). This study demonstrated that TNF‐α regulates the miR‐27a‐3p–Sfrp1 axis in osteoporosis. Overexpression of miR‐27a‐3p or knockdown of Sfrp1 was observed to promote osteogenic differentiation of BMSCs in vitro, thus providing a potential therapeutic strategy targeting miR‐27a‐3p/Sfrp1 for regenerating the bone mass.

Inflammatory cytokines such as TNF‐α, IL‐1β and IL‐6 are elevated in postmenopausal osteoporosis and have been shown to play a role in the development of the disease (Zheng et al., 1997). This study employed the OVX mouse model and confirmed that TNF‐α expression was elevated in OVX mice compared to the Sham group. Multiple mechanisms have illustrated how TNF‐α suppresses osteogenic differentiation, such as enhancing purinergic P2Y2 receptor expression, inducing semaphorin3D expression, or inhibiting Foxo1 via miR‐705 upregulation (Du et al., 2018; Liao et al., 2016; Sang et al., 2017). The expression levels of various miRNAs were dysregulated in BMSCs from the OVX mice based on the results of microarray analysis for miRNA profiling (data not shown). Particularly, we focused our study on miR‐27a‐3p. Of note, the OVX mice also had a higher serum level of IL‐1β than the Sham mice, but the treatment with TNF‐α but not IL‐1β significantly downregulated miR‐27a‐3p level in BMSCs. Therefore, we further focused on the relationship between TNF‐α treatment and miR‐27a‐3p expression during osteogenic differentiation of BMSCs in vitro.

TNF‐α initiates miRNA–mRNA signalling cascades and regulates the expression of multiple miRNAs, including miR‐27a‐3p in obstruction‐induced bladder dysfunction (Koeck et al., 2018). In another study, Wang et al. demonstrated that TNF‐α treatment enhanced lncRNA FOXD2‐AS1 expression, while FOXD2‐AS1 induced chondrocyte proliferation via sponging miR‐27a‐3p in osteoarthritis (Wang et al., 2019). The regulation of miR‐27a‐3p expression by TNF‐α could be a coincidence from other biochemical events. Whether TNF‐α directly regulates miR‐27a‐3p expression requires further investigation. Studies have demonstrated that miR‐27 is involved and plays critical roles in adipogenic and osteogenic differentiation (Lin et al., 2009; Wang & Xu, 2010). Given the fact that osteoporosis develops from the imbalance between adipogenic and osteogenic differentiation, we set out to investigate the expression pattern and functional role of miR‐27a‐3p in osteoporosis. Overexpression of miR‐27a‐3p was observed to significantly enhance osteoblast differentiation with upregulated ALP expression and increased formation of mineralized nodules, while inhibition of miR‐27a‐3p had the opposite effect. We also found that overexpression of miR‐27a‐3p enhanced consistently the expressions of osteoblast‐specific markers Runx2 and Osterix. Furthermore, miR‐27a‐3p expression peaked at day 7 during the 28‐day osteogenic differentiation of BMSCs in vitro, implicating the potential indispensable role of miR‐27a‐3p in osteoblastic bone formation. Liu et al. showed that overexpression of lncRNA MEG3 promoted osteogenic differentiation of periodontal ligament stem cells by enhancing expression of IGF1 and suppressing expression of miR‐27a‐3p (Liu et al., 2019b), which indicates a negative effect of miR‐27a‐3p on osteogenesis. Our findings are consistent with a previous report by Xu et al., showing that exosomes from C2C12 myoblasts can promote pre‐osteoblast MC3T3‐E1 differentiation to osteoblasts, and the effects of exosomes depended on the miR‐27a‐3p component, as C2C12 exosomes failed to exert these effects when miR‐27a‐3p was absent (Xu et al., 2018). These results uncover a novel regulatory pathway involving miR‐27a‐3p–Sfrp1 modulating osteogenesis in the BMSC, opening new perspectives into the pathophysiology and treatment of osteoporosis.

Bioinformatics analysis combined with experimental validation was conducted to understand the functional mechanism of miR‐27a‐3p in osteogenic differentiation and revealed that Sfrp1 was the direct target of miR‐27a‐3p. Sfrp1 was identified as being associated with bone strength and body composition (Boudin et al., 2012). Interestingly, Sfrp1 has been demonstrated to regulate cell proliferation in multiple tumours such as myeloid leukaemia, ovarian cancer and colorectal cancer (Hu et al., 2019; Huang et al., 2014; Liu et al., 2019a; Pehlivan et al., 2017). Consistent with previous findings reported by Tang et al. (2019), we demonstrated that downregulation of Sfrp1 promoted the proliferation and differentiation of BMSCs. However, other studies indicate that the expression of Sfrp1 can be regulated by other miRNAs such as miR‐1180 and miR‐144 (Hu et al., 2019; Tang et al., 2019). Similarly, Wu et al. reported that miR‐27a targeted DKK2 and Sfrp1 to promote re‐osseointegration in the regenerative treatment of peri‐implantitis (Wu et al., 2019). Thus, further investigations on the miRNAs involved in the inflammatory responses and the regulation of Sfrp1 expression during osteogenic differentiation of BMSCs are warranted to fully elucidate the roles of Sfp1 in osteoporosis development.

To illustrate the complicated regulation of Sfrp1 by different miRNAs in osteoporosis, we focused on Wnt–β‐catenin signalling. Wnt signalling is reported to play crucial roles in osteogenic differentiation, and disrupted Wnt signalling in osteoblastic‐lineage cells can result in bone formation deficiency in osteoporosis (Jing et al., 2018; Tu et al., 2012). Multiples studies have reported that the Wnt signalling suppresses osteogenic differentiation of mesenchymal stem cells (Boland et al., 2004; De Boer et al., 2004; Yuan et al., 2016). In agreement with the previous finding that Sfrp1 is an antagonist of Wnt signalling (Kawano & Kypta, 2003), we showed that the miR‐27a‐3p–Sfrp1 axis regulated the Wnt3a–β‐catenin signalling pathway in osteogenic differentiation of mouse BMSCs. However, since miR‐27‐3p might have multiple downstream targets, additional effort is needed to explore other possible signalling pathways that are regulated by the miR‐27a‐3p.

In conclusion, we demonstrated that miR‐27a‐3p was suppressed by TNF‐α in osteogenic differentiation of BMSCs and miR‐27a‐3p promoted BMSC osteogenesis via negative regulation of Sfrp1 and enhancement of the Wnt3a–β‐catenin signalling cascade. Overexpression of Sfrp1 abrogated the promotive effect of miR‐27a‐3p in osteogenic differentiation of BMSCs. These results suggest a novel regulatory axis of miR‐27a‐3p–Sfrp1 in modulating BMSC osteogenesis, which sheds new light in the clinical diagnosis and treatment of osteoporosis.

## AUTHOR CONTRIBUTIONS

Dang‐Feng Zhang designed the study and reviewed the manuscript. Xiao‐Na Jin and Xing Ma wrote the manuscript. Yu‐Sheng Qiu and Wei Ma performed the experiments. Xing Dai and Zhi Zhang analysed the data. All authors have read and approved the final version of this manuscript and agree to be accountable for all aspects of the work in ensuring that questions related to the accuracy or integrity of any part of the work are appropriately investigated and resolved. All persons designated as authors qualify for authorship, and all those who qualify for authorship are listed.

## CONFLICT OF INTEREST

The authors declared no potential conflicts of interest with respect to the research, authorship, and/or publication of this article.

## Supporting information

Statistical Summary Document

Table S1. The list and relative expression levels of predicted downstream targets of miR‐27a‐3p with online tools microT and miRDB.

## Data Availability

The datasets used and/or analysed during the current study are available from the corresponding author upon reasonable request.
